# Antibiotic Use, Self-Medication, and Antimicrobial Resistance Awareness Among Health Studies Students at the University of Mostar: A Cross-Sectional Survey

**DOI:** 10.3390/antibiotics15060609

**Published:** 2026-06-16

**Authors:** Svjetlana Grgić, Katarina Šutalo, Petrana Caktaš, Timo J. Lajunen, Mark J. M. Sullman

**Affiliations:** 1 Clinic for Infectious Disease, University Clinical Hospital Mostar, 88000 Mostar, Bosnia and Herzegovina; svjetlana.grgic@mef.sum.ba (S.G.); katarina.sutalo@fzs.sum.ba (K.Š.); petrana.caktas3@mef.sum.ba (P.C.); 2 School of Medicine, University of Mostar, 88000 Mostar, Bosnia and Herzegovina; 3 Faculty of Health Studies, University of Mostar, 88000 Mostar, Bosnia and Herzegovina; 4 Department of Psychology, Norwegian University of Science and Technology, 7491 Trondheim, Norway; 5 Department of Psychology, University of Helsinki, 00014 Helsinki, Finland; 6 School of Humanities and Social Sciences, University of Nicosia, Nicosia 1700, Cyprus; sullman.m@unic.ac.cy

**Keywords:** antibiotics, antimicrobial resistance, students, health studies, rational antibiotic use, self-medication

## Abstract

Background/Objectives: Antimicrobial resistance (AMR) is a major public health problem driven partly by inappropriate antibiotic use. Students of health studies represent future healthcare professionals with an important role in patient education, infection prevention, and antimicrobial stewardship. This study assessed knowledge, attitudes, and behaviours regarding antibiotic use and AMR among students of the Faculty of Health Studies, University of Mostar. Methods: An anonymous cross-sectional online survey was conducted in March 2025 using a self-selected convenience sample. The questionnaire was adapted from a previously published survey among Cypriot university students and distributed through student WhatsApp groups and by e-mail. Of 1113 invited students, 220 completed the survey, yielding a response rate of 19.8%. Results: During the previous 12 months, 39.5% of respondents reported antibiotic use. Most respondents reported adherence to medical instructions regarding dosage and duration of therapy, while 20.5% reported self-medication with antibiotics and 29.5% reported keeping unused antibiotics at home. Approximately 42% perceived antibiotics as easy or very easy to obtain without a prescription. Only 36.4% of respondents correctly distinguished antibiotics from other medications. Although most respondents recognised that bacteria can develop resistance, misconceptions persisted regarding humans and viruses. Differences between study programmes were observed for some attitudes and perceptions, whereas gender and year of study were not significantly associated with most responses. Conclusions: Health studies students demonstrated partial knowledge of antibiotics and AMR, together with behaviours that may contribute to inappropriate antibiotic use. Strengthened curricular content on rational antibiotic use, infection management, infection prevention, and antimicrobial stewardship appears justified. The findings are also consistent with the need to consider broader stewardship measures, including better enforcement of existing prescription-only dispensing requirements in Bosnia and Herzegovina.

## 1. Introduction

Antibiotics are among the most important classes of medicines in modern healthcare because they have enabled the effective treatment of many bacterial infections and have substantially contributed to the development of modern medicine [1]. Antimicrobial resistance (AMR) occurs when microorganisms survive or grow despite exposure to antimicrobial agents that would normally inhibit or kill them. However, antibiotics are ineffective against viral infections, and their unnecessary use in such situations may lead to adverse effects such as diarrhoea, rash, and other unwanted reactions [2]. Antibiotic allergy may manifest clinically through cutaneous reactions, organ involvement, systemic reactions such as anaphylaxis, or severe cutaneous syndromes including Stevens–Johnson syndrome and toxic epidermal necrolysis [3].

The first principle in selecting appropriate antibiotic therapy is to determine whether there is a valid indication for an antimicrobial agent. Clinical signs and symptoms of infection should be considered alongside patient-specific factors such as age, medical history, and comorbidities [4]. To promote rational use and reduce the risk of antimicrobial resistance, the World Health Organization (WHO) classifies antibiotics according to the AWaRe framework into Access, Watch, and Reserve groups, which differ in recommended availability, resistance potential, and stewardship priority [5]. Nevertheless, excessive and inappropriate use of antibiotics continues to contribute substantially to the emergence and spread of resistance among pathogenic microorganisms [6,7].

Antibiotic resistance is not solely a modern phenomenon. Resistance has been documented in bacterial isolates from glacial waters more than 2000 years old and in permafrost samples over 30,000 years old, indicating that intrinsic resistance long predates the antibiotic era [7]. The greater contemporary concern, however, is acquired resistance, which is strongly driven by excessive and inappropriate antibiotic exposure [8]. The discovery of penicillin by Alexander Fleming in 1928 transformed the treatment of bacterial infections, many of which had previously been fatal. Importantly, Fleming warned in his Nobel lecture in 1945 that misuse of penicillin would lead to the development of bacterial resistance, a warning that soon proved justified when penicillin-resistant strains emerged after the drug entered widespread use [9,10].

In response to the growing burden of antimicrobial resistance, the Global Action Plan on Antimicrobial Resistance was adopted in 2015 to coordinate international efforts to reduce and control resistance [11]. AMR is now regarded as one of the major public health challenges of the twenty-first century. It has been estimated that, by 2050, drug-resistant infections could account for up to 10,000,000 deaths annually if current trends continue [12]. At the level of the European Union and the European Economic Area, recent estimates indicate that antibiotic-resistant bacteria cause more than 800,000 infections and more than 35,000 deaths annually [13]. Similar concerns are present in Bosnia and Herzegovina (B&H). According to WHO data, the proportion of antibiotic consumption from the Access group in 2018 was 66%, exceeding the former WHO target of at least 60%; however, resistance rates for methicillin-resistant *Staphylococcus aureus*, third-generation cephalosporin-resistant *Klebsiella pneumoniae*, and penicillin-resistant *Streptococcus pneumoniae* remained above the EU and EEA average [14].

Surveillance plays an essential role in understanding antimicrobial resistance and antibiotic consumption patterns. In Europe, resistance surveillance is coordinated through the European Antimicrobial Resistance Surveillance Network (EARS-Net), which evolved from the earlier European Antimicrobial Resistance Surveillance System [15]. Despite participation in some international and regional initiatives, B&H still lacks a comprehensive national system for routine monitoring of antibiotic consumption and systematic linkage of consumption data with resistance trends [16,17]. This makes national trend analysis difficult. Available local evidence nevertheless suggests problematic patterns. For example, outpatient antibiotic consumption in the Republic of Srpska increased from 19.40 defined daily doses per 1000 inhabitants per day in 2019 to 30.80 in 2020, with notable increases in the use of penicillins, cephalosporins, and macrolides during the COVID-19 pandemic [18,19].

Knowledge and awareness are key determinants of antibiotic-related behaviour [20]. Studies assessing knowledge, attitudes, and practices have consistently shown that many individuals remain unaware of the consequences of self-medication, unnecessary antibiotic use, or premature discontinuation of therapy, all of which contribute to AMR [21]. Healthcare professionals play a central role in preserving antibiotic effectiveness through appropriate prescribing, patient education, infection prevention, and stewardship activities [22,23]. Educational interventions directed at both healthcare workers and the public are therefore essential [23,24]. Because knowledge and behaviours related to antibiotic use vary across countries, health systems, and educational settings, locally generated evidence is needed to understand context-specific gaps [25]. The legal and regulatory context of antibiotic access is also relevant. In B&H, medicines are classified according to the approved dispensing regime, including medicines issued only on a physician’s prescription, medicines issued without a prescription, and medicines used only in healthcare institutions [26]. In the Federation of B&H, where Mostar is located, medicines are prescribed and dispensed according to the dispensing regime specified in the marketing authorisation, and prescription medicines are prescribed by authorised doctors of medicine or dental medicine [27]. The Law on Pharmacy Activity also states that a pharmacist may not issue a medicine authorised for prescription-only dispensing without a prescription, and pharmacy workers who issue medicines contrary to the established dispensing regime may be subject to financial penalties [27]. In Republika Srpska, for instance, 58% of surveyed pharmacies dispensed antibiotics without a prescription, highlighting a serious public health concern in B&H [28]. These findings support the need for locally relevant research and educational initiatives aimed at promoting rational antibiotic use [29].

Students of health studies represent future healthcare professionals, and their knowledge, attitudes, and behaviours are likely to influence future prescribing, dispensing, counselling, and infection control practices. Accordingly, the aim of this study was to examine knowledge, attitudes, and behaviours related to antibiotic use and antimicrobial resistance among students of the Faculty of Health Studies, University of Mostar. Particular attention was given to students’ ability to distinguish antibiotics from other medications, their perceptions of rational and irrational antibiotic use, and their self-reported practices. We hypothesised that important gaps in knowledge regarding rational antibiotic use and AMR would be present and that these gaps would be reflected in students’ attitudes and behaviours. Within the context of Bosnia and Herzegovina, this study addresses an important local evidence gap concerning antibiotic-related knowledge and behaviour among future healthcare professionals.

## 2. Results

### 2.1. Sociodemographic Characteristics

Of the 1113 students invited to participate, 220 completed the questionnaire, corresponding to a response rate of 19.8%. The sample was predominantly female, and the mean age was 23.1 ± 5.34 years, with a range of 18–50 years (Table 1). The most represented current study programme was Physiotherapy, while Sanitary Engineering had the smallest share. Among those with work experience, the most frequently reported healthcare profession was nurse/technician, followed by physiotherapist. In terms of year of study, first-year undergraduate students and second-year graduate students were the most represented groups. Work experience in the field was reported by 33.2% of respondents.

### 2.2. Antibiotic Use, Self-Medication, and Storage of Antibiotics

During the previous 12 months, 39.5% of respondents reported antibiotic use, most commonly as a single course of therapy (Table 2). A smaller proportion reported antibiotic use two to five times, while repeated use beyond this was rare. On the item concerning the most recent occasion on which antibiotics were used, most respondents reported following a doctor’s instructions regarding dosage and duration of therapy. This item assessed self-reported adherence to medical instructions and did not independently verify prescription records for each antibiotic course. Nevertheless, 29.5% reported keeping antibiotics at home that were not currently in use. No statistically significant gender differences were observed in reported antibiotic use, adherence to medical instructions, or possession of unused antibiotics.

The results of the binary logistic regression showed that none of the examined factors were significantly associated with the use of antibiotics without a physician’s recommendation. The overall model did not reach statistical significance (χ^2^(5) = 7.25, *p* = 0.203), indicating that the included predictors together do not explain a significant proportion of the variability in the observed behaviour. The model explained between 3.2% (Cox & Snell R^2^) and 5.1% (Nagelkerke R^2^) of the variance.

Individual predictor analysis showed that year of study was associated with a slight trend toward an increased likelihood of antibiotic use without a physician’s recommendation (aOR = 1.21), although this association did not reach statistical significance (*p* = 0.091) (Table 3). Gender, study programme, and work experience also showed no significant association with the examined outcome, while the wide confidence intervals indicate relatively low precision of the estimates.

Perceived ease of obtaining antibiotics without a prescription showed a tendency toward a lower likelihood of antibiotic use without a physician’s recommendation (aOR = 0.78); however, this finding was also not statistically significant. Overall, the results suggest that the analysed demographic and educational factors were not significant predictors of self-initiated antibiotic use in the studied population.

The results of the binary logistic regression showed that none of the examined factors were significantly associated with keeping leftover antibiotics at home, as shown in Table 4. Although the model including all predictors was tested jointly, it did not reach statistical significance (χ^2^(5) = 6.11, *p* = 0.296), indicating that the analysed variables together do not explain a significant proportion of the variability in this behaviour, accounting for 2.7% (Cox & Snell R^2^) to 3.9% (Nagelkerke R^2^) of the variance. When examined individually, year of study showed a tendency toward an increased likelihood of keeping leftover antibiotics (aOR = 1.13), although this effect was not statistically significant. Similarly, gender, study programme, and work experience did not show a significant effect, while their odds ratios and wide confidence intervals indicate insufficient precision of the estimates and the absence of a clear direction of association. Perceived ease of obtaining antibiotics without a prescription showed a borderline association (aOR = 0.76, 95% CI 0.55–1.05, *p* = 0.095).

**Figure 1:**
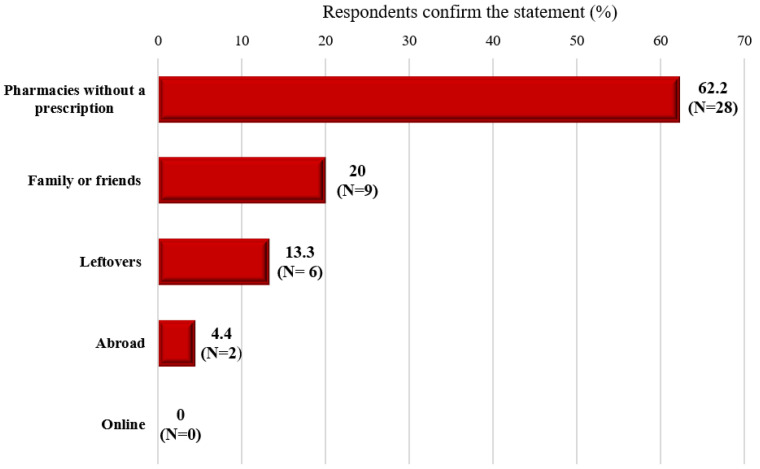
Self-reported sources of obtaining antibiotics without a physician’s recommendation.

Most respondents reported that they had never used antibiotics without a physician’s recommendation (79.5%), whereas 20.5% reported at least one form of antibiotic use without a physician’s recommendation. The most frequently reported source of antibiotics obtained without a physician’s recommendation was purchase from a pharmacy without a prescription, followed by receipt from family or friends, use of leftover antibiotics from previous treatment, and purchase abroad. No respondent reported purchasing antibiotics online (Figure 1). This item assessed self-reported use without a physician’s recommendation and should not be interpreted as verified prescription status for every antibiotic course reported during the previous 12 months. No statistically significant gender difference was found for self-medication with antibiotics (χ^2^ = 6.071; *p* = 0.194).

### 2.3. Perceived Ease of Obtaining Antibiotics Without a Prescription

**Figure 2:**
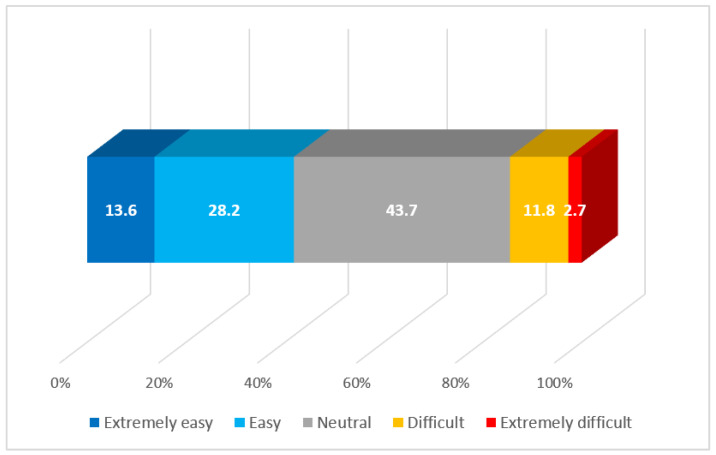
Perceived ease of obtaining antibiotics without a prescription: distribution of responses from extremely easy to extremely difficult.

Approximately 42% of respondents considered antibiotics easy (28.2%) or extremely easy (13.6%) to obtain without a prescription, whereas around 15% considered such access difficult or extremely difficult. A substantial proportion remained neutral (Figure 2). Attitudes toward antibiotic availability did not differ significantly by gender (χ^2^(4) = 2.249; *p* = 0.690) or year of study (χ^2^(16) = 13.017; *p* = 0.672). However, a statistically significant difference was observed across study programmes (χ^2^(16) = 29.175; *p* = 0.023). Among Sanitary Engineering students, approximately 79% considered antibiotics easy or very easy to obtain without a prescription, whereas 10.5% considered access difficult. Students from other programmes more often selected neutral responses, although a considerable proportion still perceived access as easy.

### 2.4. Ability to Distinguish Antibiotics from Other Medications

**Figure 3:**
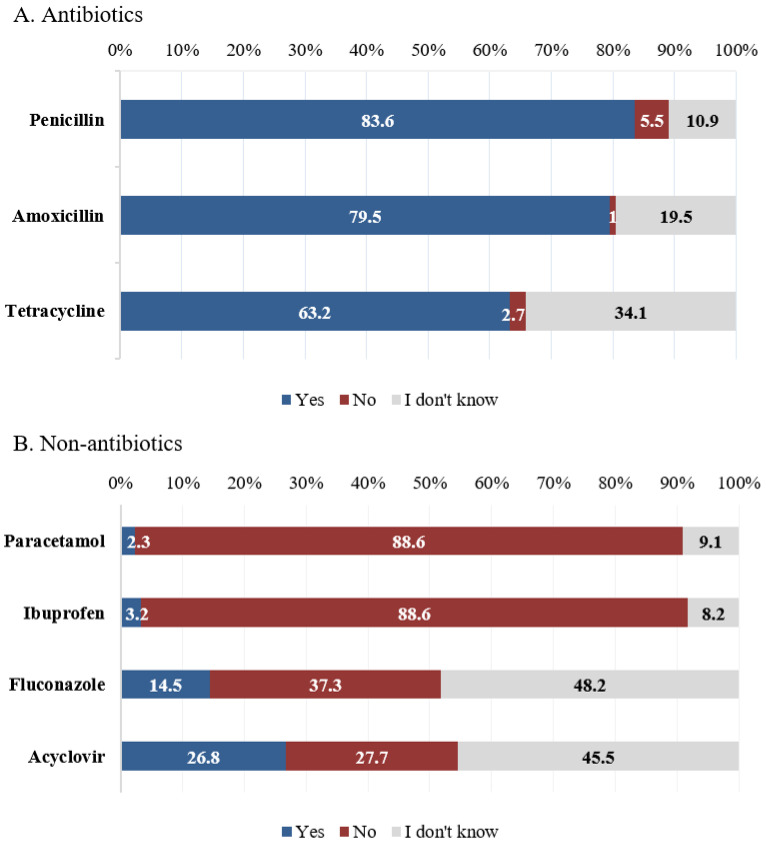
Participant responses to whether selected medicines were antibiotics: (**A**) antibiotics and (**B**) non-antibiotics.

Overall, only 36.4% of respondents correctly distinguished antibiotics from other medications. Complete correct identification was defined as correctly classifying all listed medicines as antibiotics or non-antibiotics, with “do not know” responses treated as incorrect. The highest proportion of fully correct responses was observed among students of Radiological Technology, whereas the lowest proportion was observed among Physiotherapy students. Sanitary Engineering students showed slightly better results than Physiotherapy students, while Nursing and Midwifery students achieved intermediate results. No statistically significant differences in complete correct identification were observed by gender (χ^2^(1) = 0.165; *p* = 0.684) or year of study (χ^2^(4) = 6.879; *p* = 0.142), although descriptive differences between study programmes were apparent. The detailed response distribution for each listed medicine, including yes, no, and do not know responses, is shown in Figure 3.

The results of the binary logistic regression, presented in Table 5, showed that the overall model was statistically significant (χ^2^(5) = 12.34, *p* = 0.030), indicating that the included predictors jointly contributed significantly to explaining the correct identification of antibiotics. The model explained between 5.5% (Cox & Snell R^2^) and 7.5% (Nagelkerke R^2^) of the variance in the observed outcome.

Among all examined factors, only study programme was significantly associated with the correct identification of antibiotics (aOR = 0.75, 95% CI 0.60–0.93, *p* = 0.010). This finding suggests that correct identification varied by study programme, indicating possible differences in knowledge or educational content between programmes.

On the other hand, year of study, gender, work experience, and perceived ease of obtaining antibiotics without a prescription were not significantly associated with the correct identification of antibiotics. Although year of study showed a slight trend toward an increased likelihood of correct antibiotic identification (aOR = 1.08), this association was not statistically significant. Furthermore, the wide confidence intervals for some predictors, particularly gender, indicate relatively low precision of the estimates.

### 2.5. Perceived Role of Healthcare Professionals in Preventing AMR

**Figure 4:**
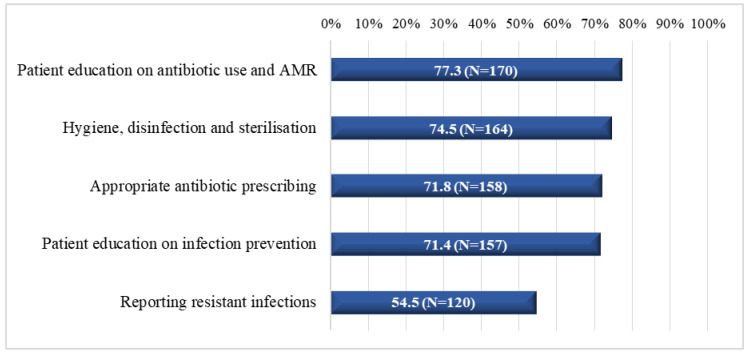
Percentage of participants endorsing statements about how healthcare professionals can help prevent antimicrobial resistance.

Respondents generally recognised that healthcare professionals have an important role in preventing and controlling AMR. More than 70% agreed that educating patients about appropriate antibiotic use and antibiotic resistance (77.3%), maintaining hand, equipment, and environmental hygiene (74.5%), prescribing antibiotics only when clinically indicated (71.8%), and educating patients about infection prevention (71.4%) are important preventive measures (Figure 4). Respondents were least likely to endorse reporting antibiotic-resistant infections to supervisory teams (54.5%) as an effective measure. When allowed to select multiple answers, 41.4% selected all proposed measures, whereas 16.3% selected only one. The number of selected statements did not differ significantly by gender (χ^2^(4) = 3.386; *p* = 0.495), but did differ by study programme (χ^2^(16) = 27.551; *p* = 0.036). Midwifery students most frequently selected all statements.

### 2.6. Knowledge and Attitudes Related to Antimicrobial Resistance

Knowledge related to the biological basis of AMR was mixed. Although 82.3% of respondents correctly recognised that bacteria can develop resistance, 83.2% also believed that humans can develop antibiotic resistance, and 36.8% attributed resistance to viruses; 21.8% were unsure about viruses (Table 6). These findings indicate persistent misconceptions regarding the mechanism of antibiotic resistance. The majority of respondents recognised that the body can overcome some mild infections without antibiotics and that leftover antibiotics should not be stored or shared. Opinions were more divided regarding the effectiveness of antibiotics for the common cold and the proper disposal of medications.

## 3. Discussion

This study provides insight into antibiotic-related knowledge, attitudes, and behaviours among students of the Faculty of Health Studies, University of Mostar. The results suggest that, although students possess some basic knowledge about antibiotics, important weaknesses remain in both conceptual understanding and reported behaviour. The most concerning findings were the low proportion of respondents who could correctly distinguish antibiotics from other medications, the persistence of self-medication and storage of unused antibiotics, and continuing misconceptions about the biological basis of antimicrobial resistance. Taken together, these findings indicate that current educational exposure may be insufficient to ensure consistently appropriate antibiotic-related knowledge and behaviour among future healthcare professionals.

The proportion of respondents reporting antibiotic use during the previous year was somewhat lower than that reported among students at the Medical University of Warsaw and among health science students in India [30,31]. Even so, the pattern of self-medication and retention of antibiotics at home is concerning. Similar problems have been reported across Southeast Europe and other settings, where non-prescription access to antibiotics contributes to irrational use [32,33]. In the present study, approximately 42% of respondents considered antibiotics easy or very easy to obtain without a prescription. This finding should be interpreted in the context of the national and entity-level dispensing framework, under which medicines are dispensed according to their authorised prescription or non-prescription status. Therefore, perceived ease of access to antibiotics without a prescription should not be interpreted as indicating that such access is formally permitted, but may reflect inconsistent enforcement, use of leftover antibiotics, informal sharing, or non-prescription dispensing in some pharmacy settings. This perception is consistent with evidence showing that perceived ease of access to antibiotics is associated with inappropriate use [34,35], and with data from Republika Srpska indicating non-prescription dispensing of antibiotics in community pharmacies [28]. Recent qualitative evidence from community pharmacists in Cyprus also shows that, even in a regulated prescription-only setting, pharmacists may face patient requests for antibiotics, attempted reuse of previous prescriptions, and use of household leftovers, while functioning as front-line antimicrobial stewards through counselling, refusal of inappropriate supply, and referral when needed [36].

Comparable patterns of self-medication have been reported in countries with different healthcare systems and levels of economic development. Studies from Lebanon [37], the United Arab Emirates [38], and Eritrea [39] documented substantial levels of self-initiated antibiotic use. Further evidence from Lebanon suggested that better knowledge may encourage more responsible behaviour [40], whereas other evidence indicates that higher educational level does not always protect against self-medication and may in some cases coexist with it [41]. In this context, the current findings support the view that knowledge is necessary but insufficient, and that behavioural and structural determinants of antibiotic use must also be addressed.

An important finding of the present study is the knowledge-behaviour discrepancy observed among respondents. Respondents often expressed support for rational antibiotic use and for the role of healthcare professionals in addressing AMR, yet problematic practices remained common. Similar discrepancies have been described in studies among medical and pharmacy students in Europe and elsewhere [42,43]. The storage of unused antibiotics at home has also been observed in other student populations and may facilitate later self-medication or sharing of medication with others [44,45,46]. Educational strategies should therefore address habits and decision-making processes rather than focusing only on factual knowledge.

Misconceptions about the common cold and about the nature of antimicrobial resistance were among the most important findings of this study. Nearly one-third of students believed that antibiotics are useful for the common cold, a misconception that has been widely documented in previous research [37,47,48]. During the COVID-19 pandemic, increased inappropriate antibiotic use and prescribing were also reported, which may have reinforced confusion about when antibiotics are indicated [18,19]. In addition, although most respondents correctly identified bacteria as capable of developing resistance, many also attributed resistance to humans and viruses. This pattern indicates confusion about the biological basis of AMR and suggests that students may not fully understand the distinction between resistant microorganisms and infected patients. Similar gaps in public understanding have been documented in B&H and across the WHO European Region [49,50].

The finding that only 36.4% of respondents correctly distinguished antibiotics from other medications is particularly concerning. Although this result is better than that reported in the Cypriot study on which the questionnaire was based, it still indicates a substantial educational deficit among future healthcare professionals [51]. Likewise, research from Nepal found that many respondents did not correctly recognise amoxicillin as an antibiotic [52]. Taken together, these findings suggest that confusion regarding basic identification of antibiotics remains common even among educated populations and should be targeted early in professional training.

Despite these shortcomings, respondents generally expressed positive attitudes toward the role of healthcare professionals in AMR prevention. Most endorsed patient education, responsible prescribing, and infection prevention as important measures, which is in line with WHO and European Commission priorities [53,54]. Evidence from Serbia and other countries suggests that educational interventions can improve knowledge and potentially support more responsible antibiotic use [55,56,57,58]. However, previous research also shows that knowledge does not automatically translate into appropriate practice, even among healthcare workers and trainees, which reinforces the need for repeated and practice-oriented stewardship education [59].

Differences between study programmes are also noteworthy, although they should be interpreted cautiously because some programme groups were small and the analyses were exploratory. Students in different programmes did not respond uniformly, particularly with respect to perceived antibiotic availability and views on the professional role in AMR prevention. These differences may reflect uneven curricular exposure to microbiology, infection prevention, pharmacology, or antimicrobial stewardship. This finding has practical implications because stewardship-related education should be embedded across all health study programmes, while examples and case-based teaching can be tailored to specific professional roles.

This programme-specific perspective is important. Sanitary engineers can contribute to reducing AMR through monitoring environmental risk pathways, supporting hygiene standards, and overseeing wastewater quality, which is increasingly recognised as relevant to the transmission of resistant microorganisms [60,61]. Nurses have a central role in infection prevention, patient education, and the promotion of rational antibiotic use [62,63,64]. Radiology departments may act as reservoirs for bacterial contamination, including multidrug-resistant organisms, which highlights the need for stronger infection prevention training among radiological technology students [65,66]. Midwives can support prudent antibiotic use during pregnancy and the perinatal period by educating women about the risks of unnecessary antibiotic use and reinforcing infection prevention principles [67]. Physiotherapists also work in settings where contaminated surfaces and equipment may facilitate pathogen transmission, making infection control competence an important professional responsibility [68].

This study has several limitations. First, it was conducted at a single faculty and used a self-selected convenience sample, which limits generalisability beyond this institution and may have produced a sample that does not fully represent health studies students elsewhere in Bosnia and Herzegovina. The sex distribution was also imbalanced, with a predominance of female respondents, which may limit the generalisability of the findings and may have reduced the ability to detect gender-based differences in antibiotic use, self-medication, and antimicrobial resistance awareness. Second, the response rate was modest, which raises the possibility of non-response bias; students with greater interest in antibiotics, public health, or academic surveys may have been more likely to participate. Third, the online self-administered format may have introduced recall bias and social desirability bias, and responses could not be independently verified against prescribing, dispensing, pharmacy, or medical records. Fourth, although the questionnaire included items on physician recommendation and self-medication, the study did not independently verify whether each antibiotic course was prescribed by a physician, dispensed without a prescription, taken from leftovers, or obtained from another informal source. Fifth, the study relied on self-reported behaviours rather than objective measures of antibiotic use. Sixth, although the questionnaire was translated and adapted to the local context by native speakers, formal back-translation and additional psychometric validation were not performed, which may affect comparability with the source instrument. Seventh, because the survey was distributed online, complete control over duplicate responses and link sharing could not be guaranteed. Finally, the cross-sectional design precludes causal inference and does not allow assessment of how knowledge and behaviours may change over time.

Future research should include students from multiple faculties and universities in B&H and should ideally compare health-related and non-health-related programmes. Studies evaluating targeted educational interventions, including stewardship-focused teaching modules, would also be valuable. Further research on antibiotic access in community pharmacies remains important, given the perceived ease of obtaining antibiotics without a prescription. Overall, the present findings suggest that improving knowledge alone may be insufficient unless educational strategies also address self-medication, antibiotic access, and the practical application of antimicrobial stewardship principles.

## 4. Materials and Methods

### 4.1. Study Design and Ethics

This anonymous cross-sectional online survey was approved by the Ethics Committee of the Faculty of Health Studies, University of Mostar (protocol code 01-313/25), and conducted in March 2025. The study was carried out in accordance with ethical principles for research involving human participants and the Declaration of Helsinki. Participation was anonymous and voluntary, and informed consent was obtained electronically before questionnaire completion.

### 4.2. Participants, Sampling, and Recruitment

The target population comprised all students enrolled at the Faculty of Health Studies, University of Mostar, during the study period. All eligible students were invited to participate; the final analytic sample was a self-selected convenience sample of respondents. The sample was neither random nor stratified. Inclusion criteria were current enrolment at the Faculty of Health Studies, University of Mostar, and voluntary participation. The only exclusion criterion was lack of consent to participate.

The survey link was distributed online through student WhatsApp groups and by e-mail. A total of 1113 students were invited to participate, of whom 220 completed the questionnaire, corresponding to a response rate of 19.8%. No financial or academic incentive was offered for participation.

### 4.3. Questionnaire

Data were collected using a questionnaire adapted from the study by Baddal et al. on knowledge, attitudes, and behaviours regarding antibiotic use among Cypriot university students [51]. Permission to adapt the questionnaire was obtained from the authors. The adaptation process included translation into Croatian and contextual adjustment of selected items for Bosnia and Herzegovina. The questionnaire was translated by study authors who are native speakers. Formal back-translation and psychometric validation were not performed.

The questionnaire consisted of 22 items divided into two parts. The first part collected sociodemographic data, including gender, age, study programme, year of study, and work experience in the field. The second part included items addressing antibiotic use, self-medication, storage of antibiotics at home, perceived availability of antibiotics without a prescription, knowledge of antibiotics and antimicrobial resistance, and perceived roles of healthcare professionals in preventing AMR. The questionnaire included items on antibiotic use during the previous 12 months, adherence to a doctor’s instructions during the most recent antibiotic use, possession of unused antibiotics at home, perceived ease of obtaining antibiotics without a prescription, and antibiotic use without a physician’s recommendation. However, the questionnaire did not independently verify prescription records or determine prescription status for every antibiotic course reported by respondents.

For the medicine-identification item, participants were asked to classify selected medicines as antibiotics or non-antibiotics. Complete correct identification was defined as correctly classifying all listed medicines, with “do not know” responses treated as incorrect. The detailed distribution of responses for each listed medicine is presented in Figure 3.

### 4.4. Data Collection and Missing Data

The questionnaire was created in Google Forms and administered online throughout March 2025. Participation was anonymous and voluntary. Before accessing the questionnaire, participants viewed study information and provided electronic informed consent.

All questions were mandatory except for the item concerning work experience in the field. Respondents who answered affirmatively to having work experience were asked to specify the occupation; otherwise, that follow-up item could be skipped. Missing data were therefore minimal and limited to this conditional item. No imputation of missing data was performed.

The survey was distributed only through channels intended for students of the Faculty of Health Studies. Eligibility was based on current student status and consent to participate. Because the survey was anonymous and distributed online, complete technical prevention of duplicate responses could not be guaranteed.

### 4.5. Statistical Analysis

Data were analysed using SPSS version 23 (IBM Corp., Armonk, NY, USA). Descriptive statistics were presented as absolute and relative frequencies for categorical variables and as mean and standard deviation for continuous variables. The chi-square test was used to examine associations between categorical variables, including gender, study programme, year of study, and students’ responses regarding antibiotic use, self-medication, perceived antibiotic availability, and AMR-related knowledge and attitudes. Binary logistic regression analyses were conducted for three binary outcomes: antibiotic use without a physician’s recommendation, keeping leftover antibiotics at home, and correct identification of antibiotics. The predictors entered into each model were year of study, gender, study programme, work experience, and perceived ease of obtaining antibiotics without a prescription. Adjusted odds ratios, 95% confidence intervals, and *p*-values were reported. Because several comparisons were performed and some subgroup sizes were small, programme-level comparisons were interpreted as exploratory. Statistical significance was set at *p* < 0.05. Microsoft Excel 2013 was used for graphical presentation of the results.

## 5. Conclusions

Students of the Faculty of Health Studies, University of Mostar, demonstrated partial knowledge regarding antibiotics and antimicrobial resistance, alongside behaviours that may contribute to inappropriate antibiotic use. The most concerning findings were the low proportion of students able to correctly distinguish antibiotics from other medications, persistent misconceptions about the biological basis of antimicrobial resistance, self-medication with antibiotics, and the storage of unused antibiotics at home. These findings support strengthening undergraduate and graduate education on rational antibiotic use, infection management, infection prevention, and antimicrobial stewardship across health study programmes. Although a single-centre survey cannot determine system-wide policy priorities, the results are consistent with the need to consider broader stewardship measures, including better enforcement of existing prescription-only dispensing requirements and improved monitoring of antibiotic consumption in Bosnia and Herzegovina.

## Figures and Tables

**Table 1:** Sociodemographic characteristics of respondents.

Variables	Total (n = 220)	
	N	%
Gender		
Male	23	10.5
Female	197	89.5
Age *	23.1 ± 5.34	
Current study programme		
Sanitary Engineering	19	8.6
Radiological Technology	39	17.7
Nursing	41	18.6
Midwifery	25	11.4
Physiotherapy	96	43.6
Year of study		
1st year undergraduate	77	35.0
2nd year undergraduate	26	11.8
3rd year undergraduate	22	10.0
1st year graduate	28	12.7
2nd year graduate	67	30.5
Work experience		
Yes	73	33.2
No	147	66.8
Healthcare profession among respondents with work experience (n = 73)		
Nurse/technician	38	52.0
Radiological technologist	7	9.6
Sanitary engineer	2	2.7
Midwife	8	11.0
Physiotherapist	18	24.7

Note: Current study programme refers to the academic programme in which respondents were enrolled. Healthcare profession refers only to the subgroup of respondents who reported work experience in the field. * Mean and standard deviation.

**Table 2:** Antibiotic use, adherence to medical instructions, and possession of unused antibiotics by gender.

	Total (n = 220)	Male (n = 23)	Female (n = 197)	Chi Square Test
	N (%) *	N (%) *	N (%) *	
*Have you used antibiotics in the past 12 months?*				χ^2^ (2) = 4.379; *p* = 0.112
Yes	87 (39.5)	11 (47.8)	76 (38.6)	
No	131 (59.5)	11 (47.8)	120 (60.9)	
I don’t know	2 (0.9)	1 (4.3)	1 (0.5)	
*How many times have you taken antibiotics in the past 12 months?*				
Never	133 (60.5)	11 (47.8)	122 (61.9)	χ^2^ (3) = 3.452; *p* = 0.327
Once	67 (30.5)	8 (34.8)	59 (29.9)	
2–5 times	17 (7.7)	3 (13.0)	14 (7.2)	
More than 5 times	3 (1.3)	1 (4.4)	2 (1.0)	
*The last time you used antibiotics, did you follow your doctor’s instructions about dosage and length of treatment?*				
Yes	202 (91.8)	21 (91.3)	181 (91.9)	χ^2^ (2) = 1.520; *p* = 0.468
No	7 (3.2)	0 (0)	7 (3.6)	
I don’t know	11(5.0)	2 (8.7)	9 (4.6)	
*Do you have antibiotics at home that you are not currently using?*				
Yes	65 (29.5)	9 (39.1)	56 (28.4)	χ^2^ (2) = 2.403; *p* = 0.301
No	137 (62.3)	11 (47.8)	126 (64.0)	
I don’t know	18 (8.2)	3 (13.0)	15 (7.6)	

* Absolute number (percentage).

**Table 3:** Binary Logistic Regression Analysis of Antibiotic Use Without Physician Recommendation.

Predictor	aOR	95% CI	*p*
Year of study	1.21	0.97–1.50	0.091
Gender	0.69	0.25–1.91	0.468
Study programme	1.01	0.78–1.31	0.938
Work experience	0.66	0.33–1.33	0.248
Ease of obtaining antibiotics	0.78	0.54–1.12	0.173

**Table 4:** Binary Logistic Regression Analysis of Factors Associated with Keeping Leftover Antibiotics at Home.

Predictor	aOR	95% CI	*p*
Year of study	1.13	0.93–1.37	0.209
Gender	0.59	0.24–1.46	0.252
Study programme	1.02	0.81–1.28	0.880
Work experience	0.93	0.49–1.74	0.818
Ease of obtaining antibiotics	0.76	0.55–1.05	0.095

**Table 5:** Binary Logistic Regression Analysis of Factors Associated with Correct Identification of Antibiotics.

Variable	aOR	95% CI	*p*
Year of study	1.08	0.90–1.30	0.390
Gender	1.22	0.46–3.19	0.692
Study programme	0.75	0.60–0.93	0.010
Work experience	0.96	0.52–1.75	0.889
Ease of obtaining antibiotics	0.98	0.72–1.33	0.874

**Table 6:** Knowledge and attitudes related to antibiotic use and antimicrobial resistance.

Statements	Yes	No	I Don’t Know
	N (%)	N (%)	N (%)
1 Bacteria can become resistant to antibiotics.	181 (82.3)	9 (4.1)	30 (13.6)
2 Viruses can become resistant to antibiotics.	81 (36.8)	91 (41.4)	48 (21.8)
3 Humans can become resistant to antibiotics.	183 (83.2)	10 (4.5)	27 (12.3)
4 Infections caused by antibiotic-resistant bacteria are on the rise in B&H.	99 (45.0)	5 (2.3)	116 (52.7)
5 Leftover antibiotics can be saved for personal use in the future or given to someone else.	27 (12.3)	153 (69.5)	40 (18.2)
6 Leftover antibiotics should be returned to the pharmacy.	64 (29.1)	72 (32.7)	84 (38.2)
7 Antibiotics help you recover faster when you have a cold.	64 (29.1)	82 (37.3)	74 (33.6)
8 The body can usually overcome mild infections on its own without antibiotics.	144 (65.5)	14 (6.4)	62 (28.2)

## Data Availability

The data presented in this study are available from the corresponding author upon reasonable request, subject to applicable ethical and privacy considerations.
